# Quasispecies of genotype 4 of hepatitis C virus genomes in Saudi patients managed with interferon alfa and ribavirin therapy

**DOI:** 10.4103/0256-4947.60515

**Published:** 2010

**Authors:** Ahmed A. Al-Qahtani, George Kessie, Damian Dela Cruz, Faleh Z. Al-Faleh, Mohammed N. Al-Ahdal

**Affiliations:** a From the Department of Biological and Medical Research, King Faisal Specialist Hospital and Research Center, Riyadh, Saudi Arabia; b From the Department of Medicine, College of Medicine, King Saud University, Riyadh, Saudi Arabia; c From the Department of Pathology and Laboratory Medicine, King Faisal Specialist Hospital and Research Center, Riyadh, Saudi Arabia; * Current address: SAIC-Frederick, National Cancer Institute at Frederick, P.O. Box B Frederick, Maryland, 21702 USA

## Abstract

**BACKGROUND AND OBJECTIVES:**

Many patients with hepatitis C virus (HCV) infection do not respond to antiviral treatment, possibly due to viral quasispecies. We aimed to investigate whether the quasispeices population could be used as a predictor of response to therapy in our patients.

**METHODS:**

The quasispecies of HCV genotype 4 (HCV-4) were studied in 25 naïve Saudi patients at zero, three, and six months following interferon alfa and ribavirin combination therapy. Hypervariable region 1 within the E2/NS1 gene of the virus was analyzed by the single-strand conformation polymorphism (SSCP) technique after amplification.

**RESULTS:**

Pretreatment DNA bands by SSCP (2-7 bands) were detected in all patients. In those who achieved a complete virological response within six months (viral load <0.2 Meq/mL; n=7), bands ranged from 2-6 (mean = 3.71±1.25). In six of these seven patients, the number of SSCP bands remained either the same or decreased sequentially. In those patients who did not respond (viral load >0.2 Meq/mL; n=18), the bands also ranged from 2-7; mean 3.77±1.73. In six of these non-responding patients, the SSCP bands remained the same or decreased sequentially. There was no significant difference between pretreatment quasispecies composition and response (*P=*·53). Two of the four patients with pretreatment high viral load and the same or decreased composition of quasispecies bands responded to the therapy.

**CONCLUSION:**

Quasispecies in our studied patients cannot be used to predict responsiveness to treatment, but may offer an explanation for failure of most HCV-4 patients to respond to interferon alfa and ribavirin therapy.

Hepatitis C virus (HCV) belongs to the genus *Hepacivirus* of the family Flaviviridae. HCV constitutes a major health problem globally and it is estimated that 170 to 200 million individuals are chronically infected with HCV worldwide, with more than one million new cases every year.^1^ HCV has a genome of nearly 9.6 kb that encodes a single large open-reading frame that yields a 3000-amino acid polyprotein. The large polypeptide is further post-translationally processed into several structural and non-structural proteins.^2^,^3^ The HCV genome contains two conserved regions at its 5' and 3' ends that are essential for translation of the polypeptide and for viral replication.^4^ HCV replicates at a very high rate resulting in a daily production of nearly 10^12^ virions, and the turnover of HCV virions is very short, reaching a maximum time of three hours.^5^,^6^ During replication, mutations in the virus genome are generated due to the limited fidelity of the viral RNA-dependent RNA polymerase. As a result, HCV circulates in an infected individual as a heterogeneous population of different, but closely related genomes, known as quasispecies.^7^,^8^

The hypervariable region 1 (HVR1) of 27 amino acids, at the N terminus of the E2/NS1 region is considered the most variable in the HCV genome with a high frequency of mutations that exhibit significant amino acid variations between different genotypes and within the same genotype, during infection.^9^ HVR1 is expressed on the surface of the virus and is thought to be the major epitope for neutralizing antibodies and for specific T cells.^10^,^11^ It has been suggested that genetic variations in the quasispecies in HVR1 are the direct result of the selective pressure of the immune response during infection.^12^,^13^ Many biological and clinical properties of the virus such as selection of the host immunity escape mutants and persistence of viral infection are attributed to the presence of quasispecies.^14^–^17^ Furthermore, HCV quasispecies may influence the progression of liver diseases and the overall clinical and histological features after interferon alfa treatment.^18^–^20^

The current FDA-approved treatment of HCV infection is a combination of ribavirin and polyethylene glycol interferon (PEG-IFN), which includes two forms namely PEG-interferon alfa-2a (PEG-INTRON) and PEG-interferon alfa-2b (PEGASYS).^21^,^22^ The attachment of PEG to interferon alfa extends its half-life and this reduces the number of injections of the drug. It was recently suggested that the quasispecies complexity of HCV may be a possible independent predictor of response to interferon alfa therapy. This conclusion was based on several studies that showed that the existence of a highly heterogeneous population of HVR1 sequences before treatment correlates with a lower rate of response to interferon alfa, independently of viral load and HCV genotype.^23^,^24^ We investigated the effects of interferon alfa and ribavirin combination therapy on the evolution of HCV quasispecies in 25 of patients at the King Khalid University Hospital, with genotype 4 (HCV-4), who were enrolled in a previous study,^25^ and determined whether the quasispecies population could be a predictor of sustained virological response in Saudi patients.

## METHODS

Patient characteristics, clinical profiles, detection, quantification (viral load), and genotyping of serum HCV RNA were reported previously.^25^ Twenty-five of these patients had left-over serum samples in our freezers that were used for this study. Each of the 25 naïve Saudi patients received ribavirin (Virazol, ICN Pharmaceuticals Inc., Costa Mesa, CA, USA) at 1000 mg daily, in two divided doses and interferon alfa-2a (Intron-A, Schering-Plough International, Kenilworth, NJ, USA) at three million units, three times a week, for six months. All 25 patients had HCV-4 infection as confirmed by an InnoLiPA HCV II Genotyping Kit (Innogenetics, Belgium), which was used according to the manufacturer's instructions. HCV RNA was extracted from 100 μL of serum, using the Q1Aamp Viral RNA Kit (QIAGEN Inc. Santa Clarita, CA, USA) according to manufacturer's instructions. cDNA synthesis was performed as previously described.^26^

Primers for nested PCR were selected from E1 to E2 genes flanking the HVR1 region of the HCV genome.^27^ The first round of amplification was carried out in a total volume of 50 μL using 20 μL of HCV cDNA preparation and 30 μL of PCR master mix (10 mM Tris-HCl, pH 8.3, 50 mM KCl, 1.5 mM MgCl_2_, 0.01% gelatin, 0.2 mM of each of the dNTPs (dATP, dGTP, dCTP, and dTTP), 40 pmoles of outer primers QP1 and QP2). The sequences of QP1 (sense) and QP2 (antisense) were: 5′-AGAATGGCCTGGGACATGATG-3′(nt 1228-1248) and 5′-CAGTGTTTAAGCTGTCATTGC-3′ (nt 1565-1585), respectively. A ‘hot start’ PCR reaction was initiated by the addition of 1.25 U of Amplitaq DNA Polymerase. PCR cycling parameters consisted of denaturation at 94°C for 1 minute, annealing at 55°C for 1 minute, and extension at 72°C for 1 minute, for 35 cycles. The second round was performed with 40 pmoles for each of the inner primers, with 2 μl of the primary product as the template. The sequences of QP3 (sense) and QP4 (antisense) were: 5′-ACTGGGGTGTCCTCGTGGG-3′ (nt1334-1352) and 5′-AACAGCAATGGGAGCTGGCA-3′ (nt 1522-1541), respectively. All primer sequences were from the GenBank database Accession Number Y11604.1. Thirty cycles of amplification were performed using the same cycling conditions as the first round except that the final extension step was increased to 7 minutes, to allow the complete formation of duplex molecules. The amplified products (10 μL) were analyzed on 2% agarose gel electrophoresis, stained with ethidium bromide (1 μg/mL), and visualized under UV illumination.

The concentration of the PCR products was determined by using the DNA Dip Stick Kit (Invitrogen, San Diego, CA). SSCP was performed as previously described.^28^ Briefly, 20 μl (15 μg) of the amplicons were mixed with equal volume of a solution containing 98% formamide, 0.05% bromophenol blue, and 2% glycerol, and denatured by heating at 94°C for 5 minutes, and immediately chilled on ice. The denatured samples were applied on 12.5% neutral polyacrylamide gels and separated at 200 V constant voltage at 25°C for 2 hours. The DNA bands were visualized by silver staining (PhastGel, Pharmacia).

The results were expressed as a mean±SD. Differences in proportions were tested by the chi-square test. Mean quantitative values were compared by the t-test. All *P* values were two-tailed and a level of less than.05 were considered statistically significant.

## RESULTS

**Figure 1:**
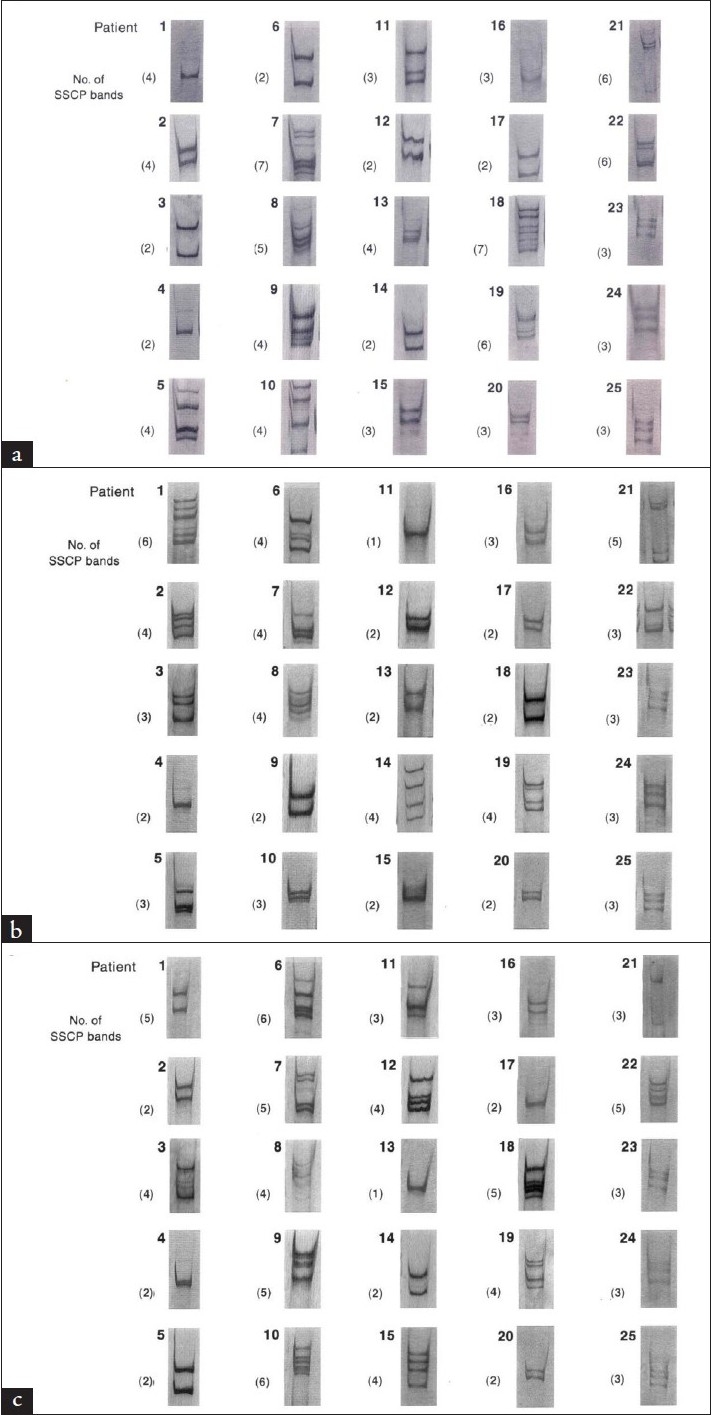
Quasispecies population in each of the 25-genotype 4, HCV-positive specimens. (a) pretreatment quasispecies population for all patients, (b) quasispecies population 3-months post-treatment, and (c) 6-months post treatment. Many bands may not be clearly seen as they were faint in the original gel due to lower concentration of the variants.

E2/NS1 fragment containing a HVR1 of HCV-4 genome was amplified from the serum samples of Saudi patients at zero, three, and six months of interferon alfa and ribavirin combination therapy. The amplified products were used for SSCP analysis, to investigate the constitution of HVR1 quasispecies in the patients. A positive virological response (responsive patients or responders) was assessed by the disappearance of the viral serum RNA (viral load <0.2 Meq/mL) as long as six months of treatment. Figures 1a, 1b, and 1c illustrate the SSCP bands obtained.

The pretreatment number of the SSCP bands in patients who responded to the therapy ranged from 2 to 6 [mean number of SSCP bands=3.71±1.25], whereas, in the non-responding patients the range of SSCP bands was 2 to 7 [mean number of SSCP bands=3.77±1.73]. No significant difference was found between the pretreatment number of SSCP bands and response (*P* =.53). Seven patients (2, 4, 10, 13, 19, 20, and 25) were considered responders, six of them (4, 10, 13, 19, 20, and 25) were with a sustained virological response, as they had no detectable levels of HCV RNA at three and six months of treatment (Table 1). However, their SSCP bands varied. In all except one (patient 10), the SSCP band either remained the same or decreased. One of these patients (patient 25) had a low virus load (< 1.0) before initiation of treatment, whereas, two patients (2 and 4) had a high virus load (>10.0). Three patients (13, 19, and 20) showed a decrease in the number of SSCP bands during the treatment period and had medium viral loads (1-10 Meq/mL) prior to initiation of treatment. Therefore, in patients who responded to interferon alfa and ribavirin therapy within six months, the HCV-4 quasispecies population in the majority of them either remained the same or decreased during the treatment period.

**Table 1:** Viral load and quasispecies population of responding patients.

Patient number	Genome copies/mL ×10^6^ (number of SSCP bands)		
	Pretreatment	3 months	6 months
2	15.19 (4)	0.219 (4)	<0.2 (2)
4	12.91 (2)	<0.2 (2)	<0.2 (2)
10	4.83 (4)	<0.2 (3)	<0.2 (6)
13	3.84 (4)	<0.2 (2)	<0.2 (1)
19	1.63 (6)	<0.2 (4)	<0.2 (4)
20	1.63 (3)	<0.2 (2)	<0.2 (2)
25	0.49 (3)	<0.2 (3)	<0.2 (3)
Mean±SD of SSCP bands	3.71 (1.25)	2.86 (0.89)	2.86 (1.68)

Eighteen of the 25 patients with viral load >0.2 Meq/mL were considered as non-responders (Table 2). Among them, four patients (16, 17, 23, and 24) showed the same SSCP band number during the treatment period. Three patients (5, 8, and 21) showed a decrease and four patients (1, 3, 6, and 12) showed an increase in the number of SSCP bands during the treatment period. In seven patients (7, 9, 11, 14, 15, 18, and 22), there were a variable number of SSCP bands in which there was a decrease at three months followed by an increase at six months and vice-versa. Among the patients in whom the number of SSCP bands either remained the same or decreased, a medium viral load (1-10 Meq/mL) prior to initiation of therapy was noted. Two (6 and 12) of the four patients who showed an increase in the number of SSCP bands had a medium pretreatment viral load, whereas, the other two patients (1 and 3) had a high pretreatment viral load (>10 Meq/mL). All patients with variable number of SSCP bands (7, 9, 11, 14, 15, 18, and 22) had a medium pretreatment viral load (1-10 Meq/mL). Statistical analysis revealed no significant difference between pretreatment quasispecies composition and the response (*P* =.53).

**Table 2:** Viral load and quasispecies population of non-responding patients.

Patient number	Genome copies/mL ×10^6^ (number of SSCP bands)		
	Pretreatment	3 months	6 months
1	40.76 (4)	4.22 (6)	14.38 (5)
3	13.07 (2)	3.20 (3)	3.50 (4)
5	9.77 (4)	<0.2 (3)	22.04 (2)
6	8.11 (2)	3.37 (4)	5.25 (6)
7	6.30 (7)	2.15 (4)	1.34 (5)
8	6.29 (5)	0.72 (4)	7.72 (4)
9	4.97 (4)	5.19 (2)	0.288 (5)
11	4.76 (3)	0.864 (1)	14.15 (3)
12	4.18 (2)	19.35 (2)	21.69 (4)
14	3.57 (2)	0.27 (4)	0.91 (2)
15	3.49 (3)	0.31 (2)	0.853 (4)
16	3.34 (3)	0.25 (3)	2.44 (3)
17	2.78 (2)	1.02 (2)	0.856 (2)
18	2.40 (7)	0.72 (2)	0.55 (5)
21	1.49 (6)	<0.2 (5)	28.96 (3)
22	1.30 (6)	3.47 (3)	1.08 (5)
23	0.56 (3)	<0.2 (3)	0.26 (3)
24	0.55 (3)	1.02 (3)	0.99 (3)
Mean±SD of SSCP bands	3.77 (1.73)	3.11 (1.23)	3.77 (1.22)

## DISCUSSION

Patients with HCV infection show many HCV genetic variants of the same genotype, known as quasispecies, due to the frequent mutation of the virus as it replicates. Persistence, resistance to therapy, and the transformation seen in these patients have long been attributed to these variants.^29^ We did not study the details and outcomes of the treatment modalities, but investigated the importance of quasispecies constituents during the therapy of HCV-4 infection, to determine if it could be used to predict the treatment outcomes in our population. The treatment has been discussed elsewhere.^25^ Quasispecies heterogeneity was shown in recent studies to be a possible independent predictor of response to interferon alfa therapy. Responsive patients typically possess less HCV quasispecies population in the pretreatment sera than do non-responsive patients.^30^,^31^ A combination regimen of interferon alfa with ribavirin was demonstrated in clinical trials to produce higher rates of sustained virological, biochemical, and histological responses, particularly in patients with a relapse.^21^,^32^ However, the role of HCV quasispecies complexity on the outcome of interferon alfa and ribavirin combination therapy for HCV-4 infected Saudi patients, has not been studied. In a previous study to assess the effect of combination therapy of interferon alfa and ribavirin on chronic HCV-4 patients, we reported a poor response among the patients, with 23% showing a sustained biochemical response and 12% showing a sustained virological response at the end of a treatment period of 24 weeks (6 months).^25^ In the present study we utilized SSCP analysis, a rapid and sensitive method for determining HCV heterogeneity, to evaluate HCV quasispecies complexity in the sera of 25 patients infected with HCV-4, before initiation of combination therapy and to assess changes in the quasispecies population after three and six months of therapy. The SSCP analysis was carried out on amplicons derived from the HVR1 region. This region is known to be highly heterogeneous in the virus genome and is therefore considered to reflect most accurately the quasispecies nature of the virus in the serum of the individual patient.^13^,^33^ Previous studies of HCV quasispecies, using SSCP and heteroduplex gel shift analysis, have identified two types of populations circulating in the serum of an infected individual.^34^,^35^ The first type consists of simple quasispecies (single HCV species) characterized by a single SSCP band; the second is comprised of complex quasispecies characterized by multiple SSCP bands. Our SSCP data showed the presence of multiple bands in the pretreatment serum of both responder and non-responder patients. Hence the pretreatment serum of all patients examined contained variable quasispecies population, although statistically no difference was found in the average number of SSCP bands between responsive and non-responsive patients. The finding that none of our patients had homogenous HCV species in the pretreatment serum is significant and appears to be a unique feature of the HCV-4 virus not related to the pretreatment viral load. Indeed, despite the fact that patients in the study group were chosen randomly, a careful examination of the virus titer in the pretreatment serum supports this observation. Of seven patients who responded to the therapy, five had a low-to-medium viral load in the pretreatment serum. Of 18 patients who were non-responders, 16 also had a low-to-medium pretreatment viral load in the serum. Our findings were in agreement with previous studies carried out on patients with HCV-1b, who were also recognized to respond poorly to interferon alfa treatment.^36^ Sakuma et al.^37^ detected multiple SSCP bands in the pretreatment sera of seven of eight patients who did not respond to interferon alfa treatment. Gonzalez-Peralta et al.^38^ also reported that among chronic hepatitis C patients treated with interferon alfa, HCV-1 (unspecified subtype) was associated with an increased number of quasispecies, resulting in poor response. Zekri et al.^39^ in Egypt recently reported the effect of HCV-4 quasispecies on the treatment outcome. However, this study involved quasispecies from a less common area of the genome for this purpose (the 5' UTR) and not the usual HVR1, and the technique used was not the universally accepted SSCP. Another study reported that quasispecies were an important predictive factor for a sustained virological response for PEG-interferon alfa and ribavirin treatment.^40^ However, the investigators worked on HCV-1 for 12 weeks and also reported that it was no better than a viral load as a predictive factor.

The presence of multiple HCV species in the pretreatment serum of HCV-4 patients could be one possible explanation for the resistance of HCV-4 virus to both the interferon alfa and interferon alfa and ribavirin combination regimen. The small number of subjects in our study does not allow for analysis of other possible factors such as side effects and histological grading, but the study did demonstrate that regardless of the presence of multiple HCV species in pretreatment serum, patients with stable viral populations or those with viral populations that decreased in complexity following treatment tend to respond to therapy. Nonetheless, the determination of quasispecies population cannot be used as a reliable predictor for effective therapy of HCV-4 infection.

## References

[1] WHO (2000). Hepatitis C Virus. Wkly Epidemiol Rec.

[2] Suzuki T, Aizaki H, Murakami K, Shoji I, Wakita T (2007). Molecular biology of hepatitis C virus. J Gastroenterol.

[3] Hellen CU, Pestova TV (1999). Translation of hepatitis C virus RNA. J Viral Hepat.

[4] Kolykhalov AA, Mihalik K, Feinstone SM, Rice CM (2000). Hepatitis C virus-encoded enzymatic activities and conserved RNA elements in the 3' nontranslated region are essential for virus replication *in vivo*. J Virol.

[5] Okamoto H, Kojima M, Okada S, Yoshizawa H, Iizuka H, Tanaka T (1992). Genetic drift of hepatitis C virus during an 8.2-year infection in a chimpanzee Variability and stability. Virology.

[6] Neumann AU, Lam NP, Dahari H, Gretch DR, Wiley TE, Layden TJ (1998). Hepatitis C viral dynamics in vivo and the antiviral efficacy of interferon-alpha therapy. Science.

[7] Escarmís C, Lázaro E, Manrubia SC (2006). Population bottlenecks in quasispecies dynamics. Curr Top Microbiol Immunol.

[8] Manrubia SC, Escarmís C, Domingo E, Lázaro E (2005). High mutation rates, bottlenecks, and robustness of RNA viral quasispecies. Gene.

[9] Ogata N, Alter HJ, Miller RH, Purcell RH (1991). Nucleotide sequence and mutation rate of the H strain of hepatitis C virus. Proc Natl Acad Sci U S A.

[10] Kaplan M, Gawrieh S, Cotler SJ, Jensen DM (2003). Neutralizing antibodies in hepatitis C virus infection: A review of immunological and clinical characteristics. Gastroenterology.

[11] Mondelli MU, Cerino A, Lisa A, Brambilla S, Segagni L, Cividini A (1999). Antibody responses to hepatitis C virus hypervariable region 1: Evidence for cross-reactivity and immune-mediated sequence variation. Hepatology.

[12] Yoshioka K, Aiyama T, Okumura A, Takayanagi M, Iwata K, Ishikawa T (1997). Humoral immune response to the hypervariable region of hepatitis C virus differs between genotypes 1b and 2a. J Infect Dis.

[13] van Doorn LJ, Capriles I, Maertens G, DeLeys R, Murray K, Kos T (1995). Sequence evolution of the hypervariable region in the putative envelope region E2/NS1 of hepatitis C virus is correlated with specific humoral immune responses. J Virol.

[14] Korenaga M, Hino K, Katoh Y, Yamaguchi Y, Okuda M, Yoshioka K (2001). A possible role of hypervariable region 1 quasispecies in escape of hepatitis C virus particles from neutralization. J Viral Hepat.

[15] Shimizu YK, Hijikata M, Iwamoto A, Alter HJ, Purcell RH, Yoshikura H (1994). Neutralizing antibodies against hepatitis C virus and the emergence of neutralization escape mutant viruses. J Virol.

[16] Kumagai N, Kaneko F, Tsunematsu S, Tsuchimoto K, Tada S, Saito H (2007). Complexity of the HVR-1 quasispecies and disease activity in patients with hepatitis C. Eur J Clin Invest.

[17] Kanazawa Y, Hayashi N, Mita E, Li T, Hagiwara H, Kasahara A (1994). Influence of viral quasispecies on effectiveness of interferon therapy in chronic hepatitis C patients. Hepatology.

[18] Farci P, Quinti I, Farci S, Alter HJ, Strazzera R, Palomba E (2006). Evolution of hepatitis C viral quasispecies and hepatic injury in perinatally infected children followed prospectively. Proc Natl Acad Sci U S A.

[19] Morishima C, Polyak SJ, Ray R, Doherty MC, Di Bisceglie AM, Malet PF (2006). Hepatitis C virus-specific immune responses and quasi-species variability at baseline are associated with nonresponse to antiviral therapy during advanced hepatitis C. J Infect Dis.

[20] Rothman AL, Morishima C, Bonkovsky HL, Polyak SJ, Ray R, Di Bisceglie AM (2005). Associations among clinical, immunological, and viral quasispecies measurements in advanced chronic hepatitis C. Hepatology.

[21] Moreno-Otero R (2005). Therapeutic modalities in hepatitis C: Challenges and development. J Viral Hepat.

[22] Foster GR (2003). Pegylated interferon with ribavirin therapy for chronic infection with the hepatitis C virus. Expert Opin Pharmacother.

[23] Le Guillou-Guillemette H, Vallet S, Gaudy-Graffin C, Payan C, Pivert A, Goudeau A (2007). Genetic diversity of the hepatitis C virus: Impact and issues in the antiviral therapy. World J Gastroenterol.

[24] Toyoda H, Kumada T, Nakano S, Takeda I, Sugiyama K, Osada T (1997). Quasispecies nature of hepatitis C virus and response to alpha interferon: Significance as a predictor of direct response to interferon. J Hepatol.

[25] Al-Faleh FZ, Aljumah A, Rezeig M, Al-Kanawi, Al-Otaibi M, Alahdal M (2000). Treatment of chronic hepatitis C genotype IV with interferon-ribavirin combination in Saudi Arabia: a multicentre study. J Viral Hepat.

[26] Al-Ahdal MN, Rezeig MA, Kessie G (1997). Genotyping of hepatitis C virus isolates from Saudi patients by analysis of sequences from PCR-amplified core region of the virus genome. Ann Saudi Med.

[27] Chamberlain RW, Adams N, Saeed AA, Simmonds P, Elliott RM (1997). Complete nucleotide sequence of a type 4 hepatitis C virus variant, the predominant genotype in the Middle East. J Gen Virol.

[28] Lee JH, Stripf T, Roth WK, Zeuzem S (1997). Non-isotopic detection of hepatitis C virus quasispecies by single strand conformation polymorphism. J Med Virol.

[29] Enomoto N, Sato C (1995). Hepatitis C virus quasispecies populations during chronic hepatitis C infection. Trends Microbiol.

[30] Xu Z, Fan X, Xu Y, Di Bisceglie AM (2008). Comparative analysis of nearly full-length hepatitis C virus quasispecies from patients experiencing viral breakthrough during antiviral therapy: Clustered mutations in three functional genes, E2, NS2, and NS5^a^. J Virol.

[31] Polyak SJ, Faulkner G, Carithers RL, Corey L, Gretch DR (1997). Assessment of hepatitis C virus quasispecies heterogeneity by gel shift analysis: Correlation with response to interferon therapy. J Infect Dis.

[32] McHutchison JG, Gordon SC, Schiff ER, Shiffman ML, Lee WM, Rustgi VK (1998). Interferon alfa-2b alone or in combination with ribavirin as initial treatment for chronic hepatitis C: Hepatitis Interventional Therapy Group. N Engl J Med.

[33] Pawlotsky JM, Pellerin M, Bouvier M, Roudot-Thoraval F, Germanidis G, Bastie A (1998). Genetic complexity of the hypervariable region 1 (HVR1) of hepatitis C virus (HCV): Influence on the characteristics of the infection and responses to interferon alfa therapy in patients with chronic hepatitis C. J Med Virol.

[34] Pawlotsky JM (2006). Hepatitis C virus population dynamics during infection. Curr Top Microbiol Immunol.

[35] de Mitri MS, Mele L, Chen CH, Piccinini A, Chianese R, D'Errico A (1998). Comparison of serum and liver hepatitis C virus quasispecies in HCV-related hepatocellular carcinoma. J Hepatol.

[36] Jardim AC, Yamasaki LH, de Queiróz AT, Bittar C, Pinho JR, Carareto CM (2009). Quasispecies of hepatitis C virus genotype 1 and treatment outcome with Peginterferon and Ribavirin. Infect Genet Evol.

[37] Sakuma I, Enomoto N, Kurosaki M, Marumo F, Sato C (1996). Selection of hepatitis C virus quasispecies during interferon treatment. Arch Virol.

[38] González-Peralta RP, Qian K, She JY, Davis GL, Ohno T, Mizokami M (1996). Clinical implications of viral quasispecies heterogeneity in chronic hepatitis C. J Med Virol.

[39] Zekri AR, El-Din HM, Bahnassy AA, Khaled MM, Omar A, Fouad I (2007). Genetic distance and heterogenecity between quasispecies is a critical predictor to IFN response in Egyptian patients with HCV genotype-4. Virol J.

[40] Salmerón J, Casado J, Rueda PM, Lafuente V, Diago M, Romero-Gómez M (2008). Quasispecies as predictive factor of rapid, early and sustained virological responses in chronic hepatitis C, genotype 1, treated with peginterferon-ribavirin. J Clin Virol.

